# An Association Study between Genetic Polymorphism in the Interleukin-6 Receptor Gene and Coronary Heart Disease

**DOI:** 10.1155/2014/504727

**Published:** 2014-05-26

**Authors:** Jiangqing Zhou, Xiaoliang Chen, Huadan Ye, Ping Peng, Yanna Ba, Xi Yang, Xiaoyan Huang, Yae Lu, Xin Jiang, Jiangfang Lian, Shiwei Duan

**Affiliations:** ^1^Ningbo Medical Center, Lihuili Hospital, Ningbo University, 57 Xingning Road, Ningbo, Zhejiang 315211, China; ^2^School of Medicine, Ningbo University, 818 Fenghua Road, Ningbo, Zhejiang 315211, China

## Abstract

The goal of our study is to test the association of IL6R rs7529229 polymorphism with CHD through a case-control study in Han Chinese population and a meta-analysis. Our result showed there is a lack of association between IL6R rs7529229 polymorphism and CHD on both genotype and allele levels in Han Chinese (*P* > 0.05). However, a meta-analysis among 11678 cases and 12861 controls showed that rs7529229-C allele was significantly associated with a decreased risk of CHD, especially in Europeans (*P* < 0.0001, odds ratio = 0.93, 95% confidential interval = 0.89–0.96). Since there is significant difference among different populations, further studies are warranted to test the contribution of rs7529229 to CHD in other ethnic populations.

## 1. Introduction

Coronary heart disease (CHD) is one of the leading causes of human deaths in the developed and developing countries such as China [1]. As a complex disease, CHD results from the interaction between genetic and environmental factors. CHD is one of the most common manifestations of atherosclerosis that is related to inflammation [2]. CHD is regarded as a chronic inflammatory disease [3] that has been shown to be associated with the response to inflammatory signaling [4].

Interleukin-6 is an inflammatory cytokine [5], whose synthesis is stimulated by its binding to* IL6R*.* IL6R* signaling activates an intracellular signaling cascade leading to the inflammatory response [6] and thus has become an important therapeutic target for prevention of CHD [7, 8].

Human* IL6R* is located on 1q21, a susceptible locus for CHD.* IL6R* rs7529229 is a T/C variation associated with both* IL6R* level and a decreased risk of CHD events in Europeans [7]. Since there is a lack of evidence concerning its role in CHD in Han Chinese, the goal of our study was to replicate the association between* IL6R* rs7529229 polymorphism and CHD in Han Chinese. In addition, we performed a meta-analysis of the available case-control studies between rs7529229 of* IL6R* gene and CHD.

## 2. Methods

### 2.1. Sample Collection

A total of 459 unrelated individuals were selected between May 2011 and November 2013 from Ningbo Lihuili Hospital, Zhejiang, China. Of these, 263 patients had CHD (males: 181; females: 82; age: 61.04 ± 8.68 years) and 196 patients were non-CHD controls (males: 98; females: 98; age: 57.76 ± 7.97 years). The patients had been examined by standardized coronary angiography according to the Seldinger method [9] and were judged by at least two independent cardiologists. In CHD cases, patients (*n* = 263) were diagnosed with the angiographic evidence that coronary artery stenosis was greater than 50% in one or more major coronary arteries [10]. Gensini scoring system was used to determine the severity of CHD [11]. A total of 196 patients, who did not have detectable coronary stenosis and atherosclerotic vascular disease, were considered as controls. All individuals had no cardiomyopathy or congenital heart, liver, or renal diseases. All the samples were Han Chinese living in Ningbo of China. The blood samples were collected by the same investigators. Blood samples were collected in 3.2% citrate sodium-treated tubes and then stored at −80°C. The study protocol was approved by the Ethics Committee of Lihuili Hospital in Ningbo and informed written consent was obtained from all subjects.

### 2.2. PCR Amplification and SNP Genotyping

**Figure 1:**
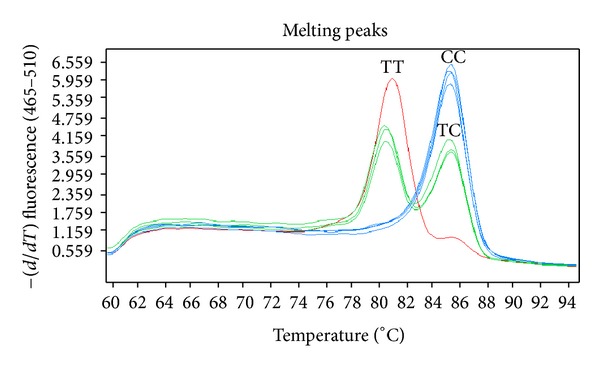
Melting temperature- (Tm-) shift method was used for SNP genotyping.

Human genomic DNA was prepared from peripheral blood samples using the nucleic acid extraction automatic analyzer (Lab-Aid 820, Xiamen, China) and was quantified using the PicoGreen dsDNA Quantification kit (Molecular Probes Inc., Eugene, OR, USA). Amplification was performed on the ABI GeneAmp PCR System 9700 Dual 96-Well Sample Block Module (Applied Biosystems, Foster City, CA, USA). Genomic DNA was subjected to polymerase chain reaction (PCR) with primers specific to* IL6R* gene. The sequences of the two allele-specific primers were 5′-GCGGCAGGGCGGCAATGTGGTCGTGGTGAGTTACCC-3′ and 5′-GATTACCGAATGTGGTCGTGGTGAGTTACCT-3′. The sequence of a reverse primer was 5^'^-TTTCTATGATTCCCTTTCACAGAGGTTTGA-3′. The reaction was performed with an initial denaturation stage at 95°C for 30 sec, followed by 40 cycles at 95°C for 30 sec, 59°C for 30 sec, and 72°C for 30 sec, and a final extension at 72°C for 30 sec. Genotyping of the PCR products was performed on the Roche LightCycler 480 Fluorescence Real-Time PCR System (Roche, Rotkreuz, Switzerland) using melting temperature shift (Tm-shift) according to the manufacturer's instructions [12, 13]. Tm-shift method uses two allele-specific primers and one reverse primer to amplify the polymorphic region encoding the targeted variant, and genotypes can be determined by inspection of a melting curve [14, 15] (Figure 1). To verify the repeatability and stability of experiment, 5% of random samples and 18 control samples (including 9 negative and 9 positive controls) were used for quality control.

### 2.3. Retrieval of Published Studies

A search was performed for the publications from 2008 to 2013 in the electronic databases (including PubMed, EMbase, Web of Science, and Cochrane Library). The search keywords included “coronary heart disease” or “coronary artery disease” or “myocardial infarction” combined with “IL6R” or “interleukin-6 receptor” or “rs7529229” or “polymorphism” and “genetic association.” We read the full-text articles to collect the relevant information. References listed on the retrieved articles and previous meta-analyses on this subject were searched to appraise other studies of potential relevance. The included studies for the meta-analysis need to be case-control design and need to have information consisting of ORs and their 95% CIs or genotyping data to measure the relative risk. Data extraction was carried out by at least two reviewers (Xiaoliang Chen and Ping Peng) on a standard protocol, and the consensus data were established by discussion. In the meta-analyses, the following data collection was included: name of the first author, publication year, country, ethnic population, study stage, numbers of individuals in the case and the control groups, OR, and 95% CI.

### 2.4. Statistical Analysis

Hardy-Weinberg equilibrium (HWE) was analyzed using the Arlequin software (v3.5) [16]. The statistical software package SPSS v.18.0 (SPSS, Chicago, IL, USA) was used for the following analyses. Continuous data were expressed as mean ± SD and Student *t*-test was employed to analyze differences between two study groups. *χ* ^2^ analysis was used to compare the categorical variables. Genotype and allele frequencies between CHD cases and healthy controls among the different subgroups were compared using *χ* ^2^ test. Association between Gensini scores and rs7529229 was compared using linear regression test. Mann-Whitney test was used for the association of Gensini scores with rs7529229 under the dominant and the recessive models. The power of the study was estimated by the Power and Sample Size Calculation software (v3.0.43) [17]. Meta-analysis was performed by Stata software version 11.0 (Stata Corporation, College Station, TX) and in accordance with Stroup's study [18]. Heterogeneity of the studies was evaluated by the *I* ^2^ statistic at the significant level of 0.05. The combined odds ratios (ORs) along with their 95% confidence intervals (CIs) were assessed with inverse-variance fixed-effect model (subtotal *I* ^2^ = 20.4%, *P* = 0.274; overall *I* ^2^ = 12.5%, *P* = 0.441) [19]. Subgroup meta-analysis was performed by ethnicity. Sensitivity analysis was conducted by omitting each study in turn. Funnel plots and Egger regression tests [20] were used to estimate the publication bias. A two-sided *P* < 0.05 was considered to be statistically significant.

## 3. Results

### 3.1. Basic Characteristics of the Study Population

As shown in Table 1, the prevalence of essential hypertension (EH), diabetes mellitus (DM), and smoking history was significantly higher in CHD patients than controls (*P* < 0.05). However, there were no significant differences between the two groups for a series of biochemical parameters, including high-density lipoprotein cholesterol (HDL-C), triglycerides, low-density lipoprotein cholesterol (LDL-C), and total cholesterol.

### 3.2. Genotype and Allele Distribution of rs7529229 in CHD Cases and Controls

Genotype distribution of rs7529229 in both CHD cases and controls met HWE (Table 2). Genotype analysis of rs7529229 did not reveal significant difference between CHD cases and non-CHD controls (*χ* ^2^ = 0.61, *P* = 0.73). The allelic distribution of rs7529229 did not differ between CHD cases and non-CHD controls (*P* = 0.43, OR = 0.90, 95% CI = 0.69–1.17, Table 2). We further examined the roles of rs7529229 in males and females separately. However, no significant differences between cases and controls were observed in male and female subgroups (Table 2). In addition, we also performed an age-stratified analysis to investigate whether age influenced the contribution of rs7529229 to the risk of CHD. Again, no significant differences between CHD cases and controls were observed in all age-stratified subgroups (Table 3).

### 3.3. Stratified Analyses of rs7529229 between Cases and Controls by Smoking History or Status of Hypertension or Diabetes

Since smoking history, hypertension, and diabetes are risk factors of CAD [21], we further performed stratified association tests by the above three variables. Our results showed that there were no significant differences in the distribution of genotype and allele of rs7529229 between cases and controls (Table 4).

### 3.4. Association of rs7529229 with the Severity of Coronary Lesions

A linear regression test of the means of Gensini scores with rs7529229 genotype did not show a statistically significant correlation (Table 5). And there was no significant association between Gensini scores and rs7529229 under the dominant (*Z* = −0.38, *P* = 0.69, Table 5) and the recessive models (*Z* = −0.50, *P* = 0.61, Table 5).

### 3.5. Meta-Analysis of rs7529229 with CHD in Different Populations

**Figure 2:**
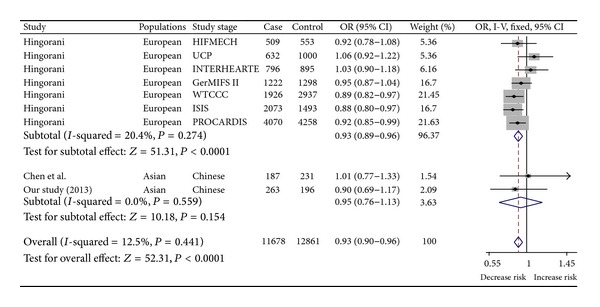
Meta-analysis of ten association studies of rs7529229 with CAD.

**Figure 3:**
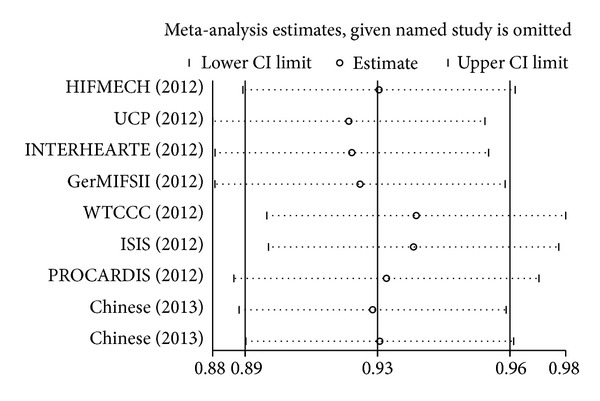
Sensitivity analysis for the association between* IL6R* rs7529229 polymorphism and CHD risk.

**Figure 4:**
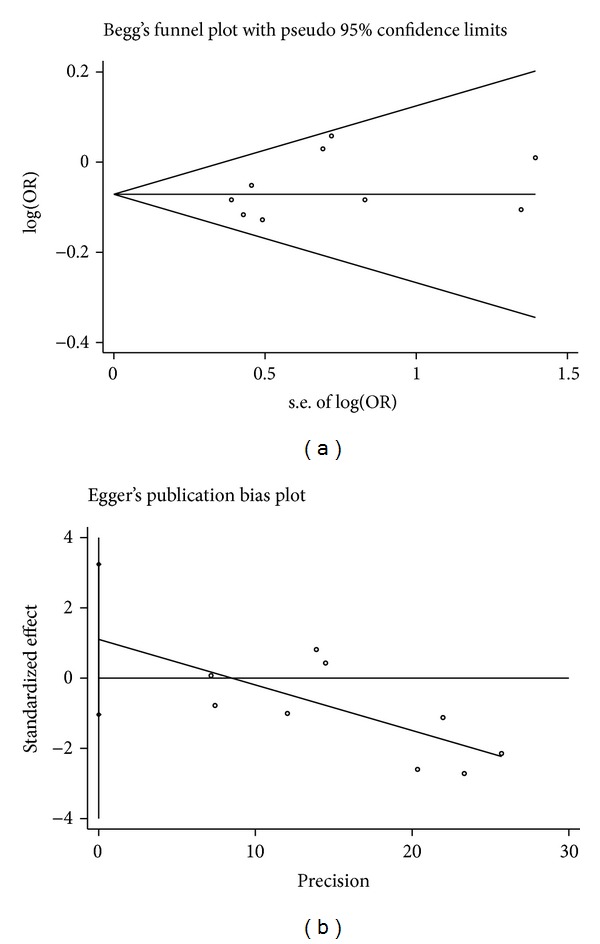
Begg's funnel plot and Egger's regression plot of 11 association tests between rs7529229 and CHD.

A total of 41 studies were selected initially. After reading the full text of these articles, 9 eligible studies were harvested for the current meta-analysis of the association of rs7529229 with CHD [7, 22, 23]. Details of articles in the meta-analysis are shown in Figure 2. Our meta-analysis comprised 11,678 CHD cases and 12,861 controls from two ethnic populations (Europeans and Asians). No significant heterogeneity was found in this meta-analysis (*P* = 0.441, *I* ^2^ = 12.5%). Our result suggested that rs7529229-C allele was associated with CHD risk. A future subgroup meta-analysis showed that rs7529229 of* IL6R* gene was a protective factor of CHD, especially in Europeans (*P* < 0.0001, OR = 0.93, 95% CI = 0.89–0.96). Sensitivity analyses were repeatedly conducted when each particular study was omitted. As shown in Figure 3, the results were not altered with pooled ORs ranging from 0.92 to 0.94 for the meta-analysis in Europeans and Asians. There was no visual publication bias in Begg's funnel (*P* = 0.25) and Egger's regression plots (*P* = 0.251, Figure 4).

## 4. Discussion

In the present study, we aim to replicate previous significant association between* IL6R *rs7529229 polymorphism and the risk of CHD in Han Chinese. Our study analyzed the association of rs7529229 with both CHD susceptibility and its severity. We also explored the stratified association of rs7529229 with CHD, though we failed to observe significant associations between* IL6R *rs7529229 and the risk of CHD. The results of our study were inconsistent with the recent findings from a large study of European samples [7]. We speculated that the discrepancies might be due to ethnic difference in the prevalence of this SNP. In addition, a power calculation showed that our case-control study only had a 12.2% power to detect a relative risk of rs7529229 at a significant level of 0.05, suggesting that a lack of power was likely to explain our failure to find a significant association.

Our meta-analysis, including a total of 11,678 cases and 12,861 controls, examined the association between the rs7529229 polymorphism and CHD risk. We found that rs7529229 of* IL6R* gene was associated with the risk of CHD. A further subgroup analysis by race showed that rs7529229 of* IL6R* gene was a protective factor of CHD, especially in Europeans. Our study is the first association test between rs7629229 and CHD in the Chinese population. We carried out sensitivity analysis to assess the stability of this meta-analysis. Removal of each study did not alter the conclusion of the CHD risk, suggesting the reliability of these results. Meta-analysis can dramatically increase the power of association test through the combination of the data from various studies. For example, the power of some studies in the current meta-analysis is moderate (HIFMECH: 60.1%; UCP: 55.3%; INTERHEARTE: 32.2%; GerMIFSII: 65.2%; Chen et al.: 11.8%).

There are several limitations in our study. Firstly, the power of our case-control study only reached 12.2% at alpha level of 0.05, so we could not exclude the possibility of lack of power in our study mainly due to the relatively small sample size. Secondly, only one polymorphism of* IL6R* was investigated in the present study. According to the report in dbSNP, there were at least 256 SNPs on the* IL6R *gene locus. Therefore, the results of* IL6R* rs7529229 might not stand for the rest of the* IL6R* SNPs. Thirdly, we only searched the literatures in Chinese for the eligible research included in the meta-analysis. Meanwhile, case-control studies with negative results were more likely to be unpublished. Potential language and publication bias might exist in the meta-analysis.

In conclusion, our meta-analysis has established a strong contribution of rs7529229-C allele to reduced risk of CHD, especially in Europeans, although our case-control study is unable to find association of the* IL6R* with the risk of CHD. Further investigation on other SNPs on the gene is warranted to validate our findings in the Chinese population.

## Figures and Tables

**Table 1:** Basic characteristics of the study population.

	CHD (*n* = 263)	Controls (*n* = 196)	*P*
Male, *n* (%)	182 (69.2%)	98 (50%)	<0.001
Smoking, *n* (%)	111 (43.5%)	53 (27.3%)	<0.001
Hypertension, *n* (%)	161 (63.1%)	100 (51.5%)	0.014
Diabetes, *n* (%)	53 (20.7%)	12 (6.1%)	<0.001
Mean age, years	61.04 ± 8.68	57.76 ± 7.97	<0.001
LDL-C (mmol/L)	2.54 ± 0.94	2.49 ± 0.89	0.600
Total cholesterol (mmol/L)	4.33 ± 1.10	4.28 ± 1.01	0.620
HDL-C (mmol/L)	1.06 ± 0.30	1.12 ± 0.29	0.048
Triglycerides (mmol/L)	1.64 ± 1.06	1.50 ± 0.86	0.125

**Table 2:** Genotype and allele distribution of rs7529229 in CHD cases and controls.

Group	Genotype (TT/TC/CC)	*χ* ^2^	*P* (df = 2)	HWE	Allele (*T*/*C*)	*χ* ^2^	*P* (df = 1)	OR (95% CI)
All CHD cases (*n* = 263)	77/133/53			0.80	287/239			
All controls (*n* = 196)	63/98/35	0.61	0.73	0.88	224/168	0.60	0.43	0.90 (0.69–1.17)
Female CHD cases (*n* = 82)	30/35/17			0.26	95/69			
Female controls (*n* = 98)	34/47/17	0.58	0.74	1.00	115/81	0.02	0.88	0.97 (0.63–1.47)
Male CHD cases (*n* = 181)	47/98/36			0.29	192/170			
Male controls (*n* = 98)	29/51/18	0.43	0.80	0.68	109/87	0.33	0.56	0.90 (0.63–1.27)

**Table 3:** Post hoc analysis of rs7529229 with the risk of CHD in different age subgroups.

Age group	Genotype (TT/TC/CC)	*χ* ^2^	*P* (df = 2)	HWE	Allele (*T*/*C*)	*χ* ^2^	*P* (df = 1)	OR (95% CI)
≤55 CHD cases (*n* = 70)	19/37/14			0.80	75/65			
≤55 controls (*n* = 71)	25/36/10	1.49	0.47	0.80	86/56	1.40	0.23	0.75 (0.46–1.20)
55–65 CHD cases (*n* = 95)	23/50/22			0.68	96/94			
55–65 controls (*n* = 82)	25/39/18	0.89	0.64	0.82	89/75	0.49	0.48	0.86 (0.56–1.30)
≥65 CHD cases (*n* = 98)	35/46/17			0.83	116/80			
≥65 controls (*n* = 43)	13/23/7	0.54	0.76	0.75	49/37	0.12	0.72	1.09 (0.65–1.82)

**Table 4:** The stratified association analysis of rs7529229.

Group	Risk factor of CHD	Genotype (TT/TC/CC)	*χ* ^2^	*P*	*T*/*C*	*χ* ^2^	*P*
CHD (*n* = 115)	Smoking	31/61/23			123/107		
Control (*n* = 55)	Smoking	15/31/9	0.339	0.844	61/49	0.117	0.732
CHD (*n* = 148)	No smoking	46/72/30			164/132		
Control (*n* = 141)	No smoking	48/67/26	0.339	0.844	163/119	0.337	0.561
CHD (*n* = 165)	Hypertension	48/86/31			182/148		
Control (*n* = 102)	Hypertension	36/44/22	2.061	0.375	116/88	0.15	0.699
CHD (*n* = 98)	No hypertension	29/47/22			105/91		
Control (*n* = 94)	No hypertension	27/54/13	2.789	0.248	108/80	0.583	0.445
CHD (*n* = 57)	Diabetes	16/27/14			59/55		
Control (*n* = 14)	Diabetes	6/5/3	1.178	0.555	17/11	0.725	0.394
CHD (*n* = 206)	No diabetes	61/106/39			228/184		
Control (*n* = 182)	No diabetes	57/93/32	0.191	0.909	207/157	0.183	0.669

**Table 5:** Association tests of Gensini scores and CHD.

Genotype	Gensini score (mean/SD/median)	*F*/*Z*	*P* (df = 1)
TT (*n* = 77)	56.12/56.08/35.5		
TC (*n* = 133)	48.67/43.67/33.0		
CC (*n* = 53)	46.60/43.64/36.0	0.30	0.85
Recessive model			
TT + TC (*n* = 210)	51.40/48.59/35.2		
CC (*n* = 53)	46.60/43.64/36.0	−0.50	0.61
Dominant model			
TC + CC (*n* = 186)	48.08/43.56/34.5		
TT (*n* = 77)	56.12/56.08/35.5	−0.38	0.69
